# Generation of novel lipid metabolism-based signatures to predict prognosis and immunotherapy response for colorectal adenocarcinoma

**DOI:** 10.1038/s41598-024-67549-x

**Published:** 2024-07-26

**Authors:** Yi Wang, Jun Yao, Zhe Zhang, Luxin Wei, Sheng Wang

**Affiliations:** 1 Department of Oncology and Hematology, Suzhou Kowloon Hospital, Shanghai Jiao Tong University School of Medicine, Suzhou, 215127 China; 2 Department of General Surgery, The Fourth Affiliated Hospital of Soochow University, Suzhou, 215127 China

**Keywords:** Colorectal adenocarcinoma, Metabolism, Epithelial-mesenchymal transition, Prognosis, Immunotherapy, Chemotherapy, Single cell, Cancer, Computational biology and bioinformatics

## Abstract

Lipid metabolism reprogramming involves in epithelial-mesenchymal transition (EMT), cancer stemness and immune checkpoints (ICs), which influence the metastasis of cancer. This study aimed to generate lipid metabolism-based signatures to predict prognosis, immunotherapy and chemotherapy response for colorectal adenocarcinoma (COAD). Transcriptome data and clinical information of COAD patients were collected from the cancer genome atlas (TCGA) database. The expression of EMT-, stem cell-, and IC-related genes were assessed between COAD and control samples. Modules and genes correlated EMT, ICs and stemness signatures were identified through weighted gene co-expression network analysis (WGCNA). Prognostic signatures were generated and then the distribution of risk genes was evaluated using single-cell RNA sequencing (scRNA-seq) data from GSE132465 dataset. COAD patients exhibited increased EMT score and stemness along with decreased ICs. Next, 12 hub genes (PIK3CG, ALOX5AP, PIK3R5, TNFAIP8L2, DPEP2, PIK3CD, PIK3R6, GGT5, ELOVL4, PTGIS, CYP7B1 and PRKD1) were found within green and yellow modules correlated with EMT, stemness and ICs. Lipid metabolism-based prognostic signatures were generated based on PIK3CG, GGT5 and PTGIS. Patients with high-risk group had poor prognosis, elevated ESTIMATEScore and StromalScore, 100% mutation rate and higher TIDE score. Samples in low-risk group had more immunogenicity on ICIs. Notably, PIK3CG was expressed in B cells, while GGT5 and PTGIS were expressed in stromal cells. This study generates lipid metabolism-based signatures correlated with EMT, stemness and ICs for predicting prognosis of COAD, and provides potential therapeutic targets for immunotherapy in COAD.

## Introduction

Globally, colorectal cancer (CRC) is the most common gastrointestinal malignancy with an estimated 1.9 million new diagnosed cases, leading to 935,000 deaths as reported by Global Cancer Statistics 2020^1^. Recently, it has been projected that the prevalence of CRC will increase to 3.2 million cases in 2040, and China is regarded to be one of the countries with the highest incidence rate within the following 20 years^2^. Colorectal adenocarcinoma (COAD) originates from colorectal mucosa epithelial cells and is the predominant diagnostic type of CRC, accounting for over 90% of total cases^3^. The 5-year survival rate of COAD patients with early stage is favorable, however, patients with metastasis or advanced stage exhibit a survival rate of 11%^4^. The therapeutic strategies are decided depending on the classical TNM stage system. Unfortunately, the treatment effectiveness and survival rates remain different for COAD patients, which may probably attribute to high heterogeneity in patients with COAD^5^. Therefore, personalized treatments and prognostic prediction are needed in clinic for COAD patients.

Tumor metastasis is a complex and life-threatening process that results in dismal prognosis and unfavorable immunotherapy response of cancer patients. Epithelial-mesenchymal transition (EMT) process has been recognized as a key event to enhance migration and invasion of stationary tumor cells^6^, and activated EMT is linked to resistance to chemotherapy or immunotherapy. Compelling evidence has revealed that EMT is mediated by the tumors microenvironment (TME), especially by hypoxic conditions in TME in COAD^7,8^. Besides, recent study has deciphered that EMT process is correlated with the increased expansion of cancer stem cells and cells with stem-like properties, leading to metastasis and therapy resistance^7^. Xu has demonstrated that co-operation of cancer stemness, immune cells and EMT can forecast clinical outcomes in CRC patients^9^.

In cancer cells, fundamental changes in cellular metabolism often occur, offering biochemical basis and leading to the occurrence and malignancy of tumors. Abnormality of lipid metabolism in tumor cells enhances the synthesis of membranous lipid and the production of bioactive lipids, therefore leading to EMT program^10^. Excessive FABP4 enhances the migration and invasion of colon cancer cells through increasing the transport of fatty acids^11^. Also, HSPC111 derived from exosome promotes metastasis of CRC to liver via lipid metabolism reprogramming in cancer-associated fibroblasts^12^. Meanwhile, altered lipid metabolism contributes to and maintains the properties of cancer stem cells^13^ and also involves in activating oncogenic signaling pathways, such as Wnt/β-catenin and Hippo/YAP pathways^14^. However, lipid metabolism-based signature in COAD remains poorly elucidated. Hence, this study generated lipid metabolism-based prognostic signatures in COAD and validated the risk genes in scRNA-seq data, which shed novel insights into underlying mechanism of lipid metabolism in COAD and also provided a method to forecast prognosis, immunotherapy and chemotherapy response for COAD patients.

## Results

### Assessment of tumor signatures using ssGSEA scores

**Figure 1:**
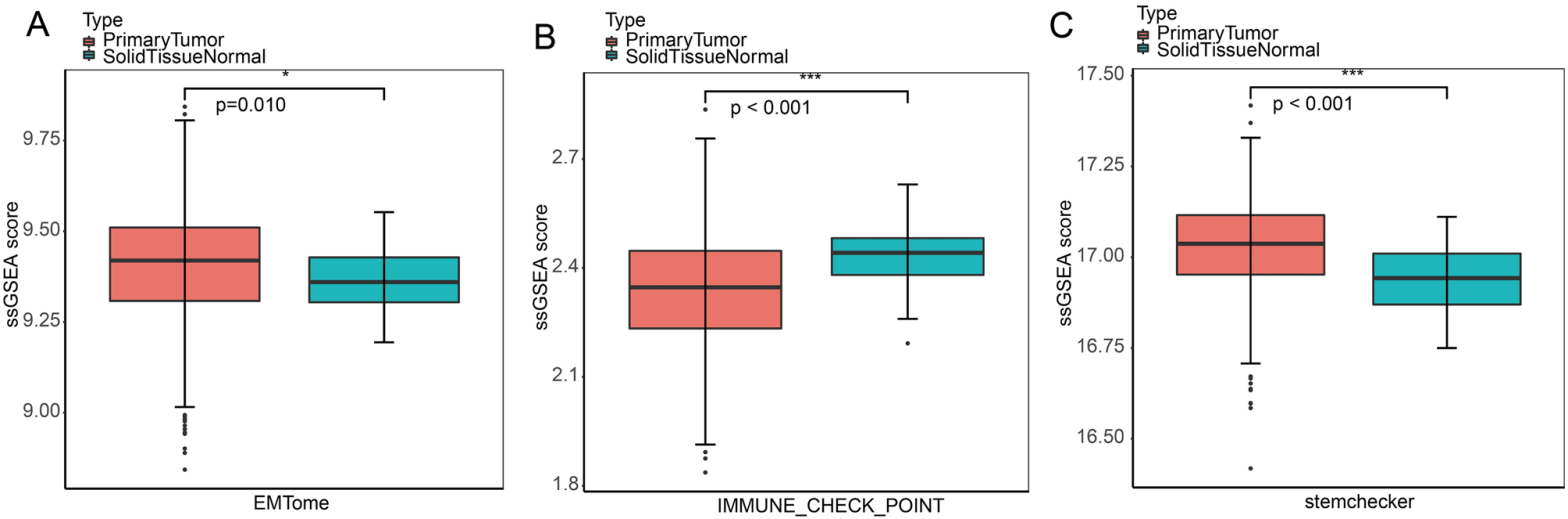
Assessment of tumor signatures in TCGA dataset. (**A**–**C**) ssGSEA scores for EMT, stemness and immune checkpoints in primary COAD samples and normal samples.

To investigate the tumor signatures, ssGSEA was used to score the EMT-related genes, stem cell-related gene and IC genes in COAD and normal samples. As shown in Fig. 1A–C, primary tumor samples possessed significant increased EMT score (*P* = 0.010) and stemness score (*P* < 0.001) than that of normal samples, while the ssGSEA score for ICs was remarkably decreased in primary tumor samples compared with normal samples (*P* < 0.001). Those data indicated that primary tumor samples maybe presented EMT, acquired characteristics of CSCs and escaped immune killing.

### Identification of co-expression modules and hub genes using WGCNA

Based on 742 lipid metabolism-related genes, WGCNA was performed to identify co-expression modules. Sample cluster analysis was conducted and the height cutoff value was set at 30 to detect outliers. A total of 65 outliers were excluded from this study (Supplementary Fig. 1A). Next, the soft threshold power at 4 with an R^2 > 0.8 is selected and 12 gene co-expression modules were identified (Supplementary Fig. 1B**,**C). Supplementary Fig. 1D displayed the eigengene adjacency heatmap that indicated that the green module, the yellow module and some other modules were adjacent.

**Figure 2:**
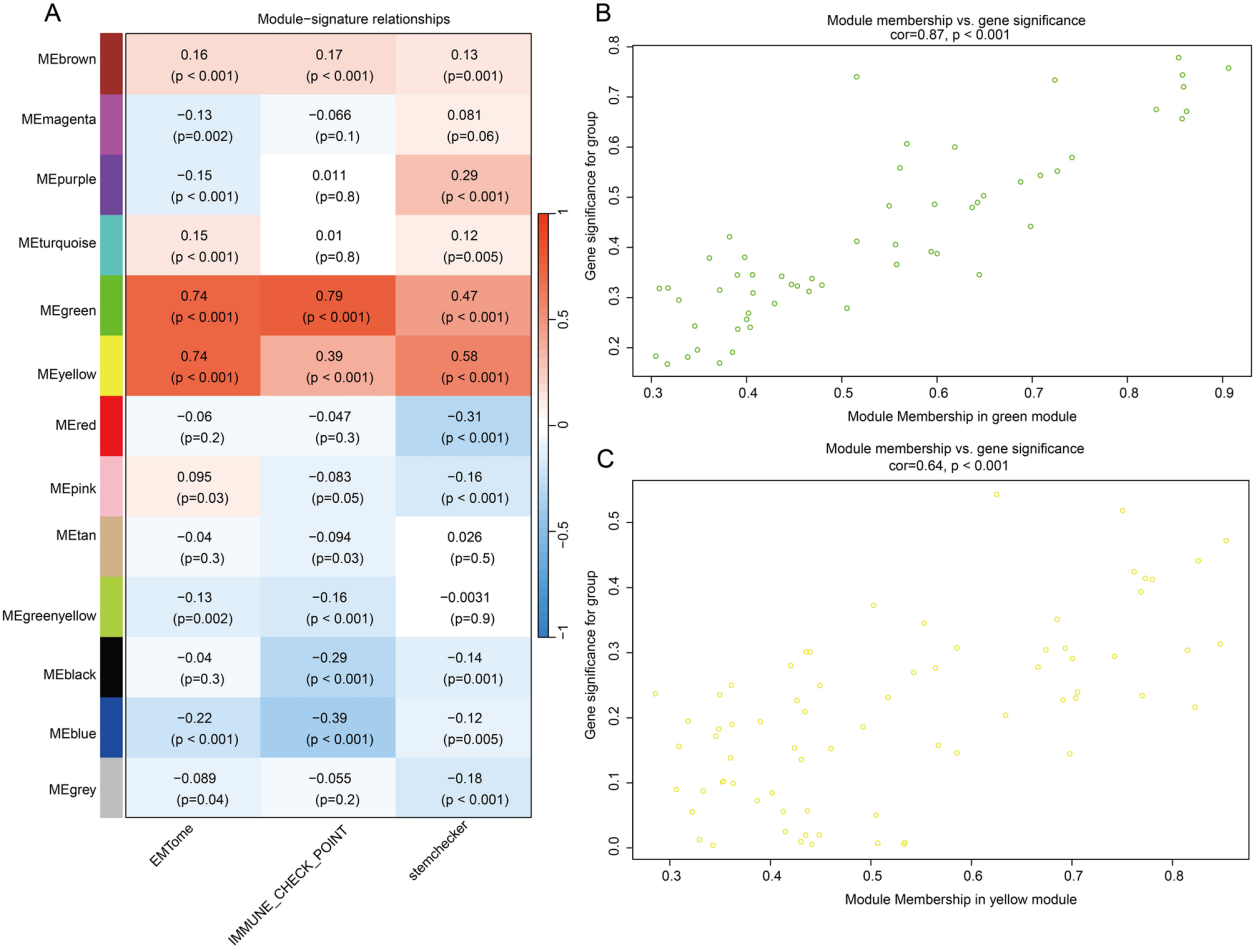
Selection of modules associated with tumor signatures. (**A**) The relationships between12 modules and tumor signatures (EMT, immune checkpoints and stemness). (**B**,**C**) Module membership analysis for green module and yellow module.

Subsequently, the module-signature relationship was evaluated (Fig. 2A). The green model was significantly correlated with EMT (cor = 0.74, *P* < 0.001), ICs (cor = 0.79, *P* < 0.001) and stemness (cor = 0.47, *P* < 0.001); also, the yellow module was correlated with EMT (cor = 0.74, *P* < 0.001), ICs (cor = 0.39, *P* < 0.001) and stemness (cor = 0.58, *P* < 0.001) of COAD. Then, the green module and yellow module were selected for module membership analysis (Fig. 2B,C). In the green module, the module membership was highly correlated with gene significance (cor = 0.87, *P* < 0.001); in the yellow module, the module membership had a significant correlation with gene significance (cor = 0.64, *P* < 0.001), indicating that the green module and yellow module were suitable for identifying hub genes. Based on intramodular connectivity |kME|> 0.8, a total of 12 hub genes (PIK3CG, ALOX5AP, PIK3R5, TNFAIP8L2, DPEP2, PIK3CD, PIK3R6, GGT5, ELOVL4, PTGIS, CYP7B1 and PRKD1) were found within the green module and yellow module.

### Generation of prognostic signatures and survival analysis

**Figure 3:**
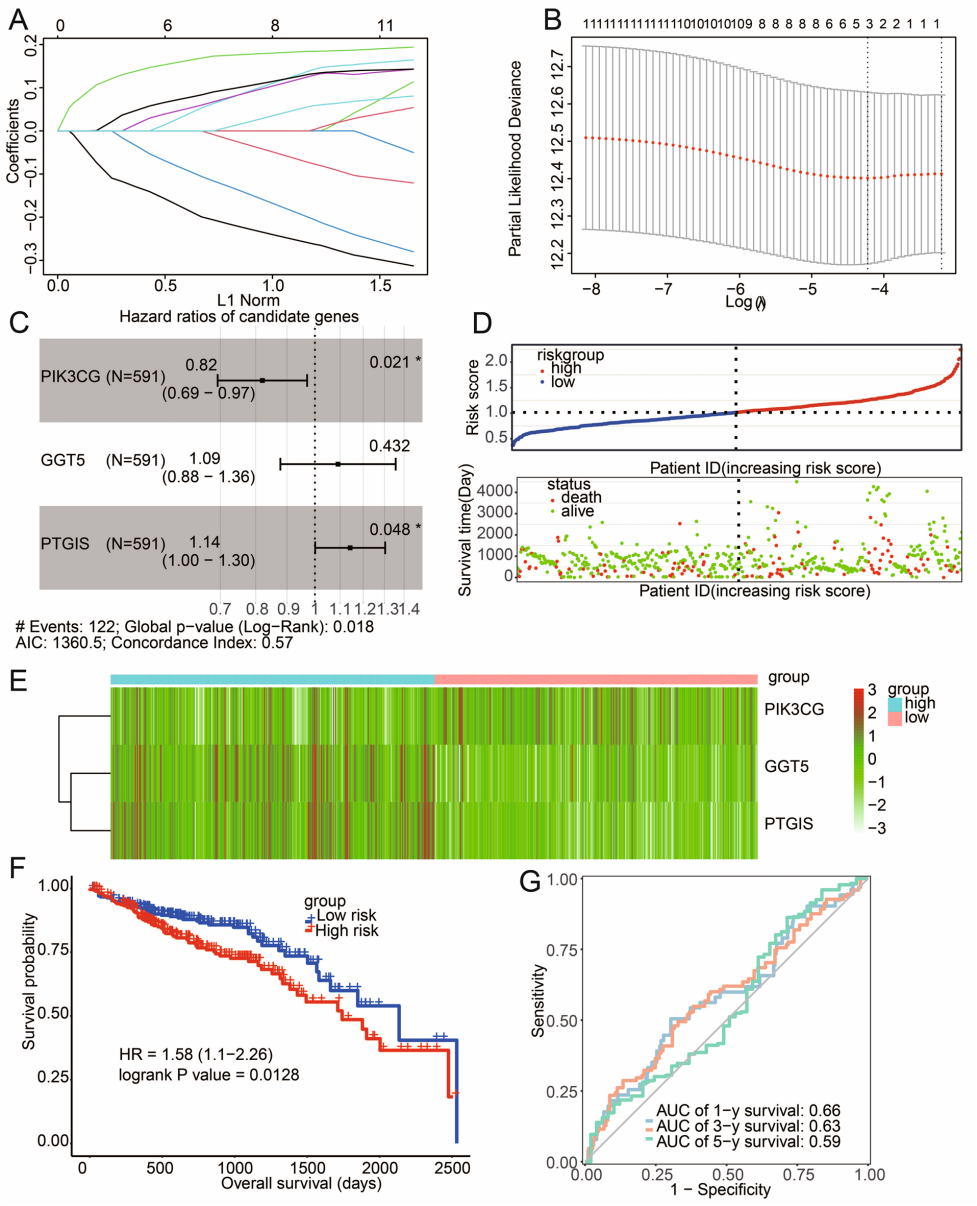
Generation of prognostic model and survival analysis. (**A**) Determination of risk genes using LASSO. Independent variable coefficients changed with lambda increase. (**B**) The partial likelihood deviance of ten-fold cross validation. (**C**) Three genes (PIK3CG, GGT5 and PTGIS) were determined as risk genes. (**D**) Distribution of risk score and the status of patients. (**E**) The expression patterns of PIK3CG, GGT5 and PTGIS in patients with high- and low-risk. (**F**) Survival analysis using Kaplan–Meier curves in TCGA dataset. G, ROC curves with 1 year AUC of 0.66, 3-year AUC of 0.63 and 5-year AUC of 0.59.

LASSO Cox regression was utilized to determine the risk genes for prognostic model construction. With the gradual increase of lambda, the number of independent variable coefficients tending to zero increased gradually (Fig. 3A). Ten-fold cross-validation was utilized to calculate partial likelihood deviances (Fig. 3B). The optimal lambda = 0.01463613. Three genes (PIK3CG, GGT5 and PTGIS) were determined for further analysis (Fig. 3C). The risk score was calculated according to the formula: Risk score = −0.19820*PIK3CG + 0.08726*GGT5 + 0.13305*PTGIS. Figure 3D revealed that the number of death was major in high-risk group. The expression of PIK3CG was decreased in high-risk group while the expression of GGT5 and PTGIS were increased in high-risk group (Fig. 3E). Further survival analysis in TCGA dataset deciphered that patients in high-risk group had dismal prognosis than that of patients in low-risk group (*P* = 0.0128) (Fig. 3F) with 1 year AUC of 0.66, 3-year AUC of 0.63 and 5-year AUC of 0.59 (Fig. 3G). Furthermore, In the KMPLOT database (https://kmplot.com), COAD samples were selected for survival analysis. The result showed that COAD patients with high expression of PIK3CG (*P* = 0.033), or PTGIS (*P* = 0.00032), and low expression of GGT5 (*P* = 0.047) have a low overall survival rate (Supplementary Fig. 2).

### Analysis of mutation characteristics between risk groups

**Figure 4:**
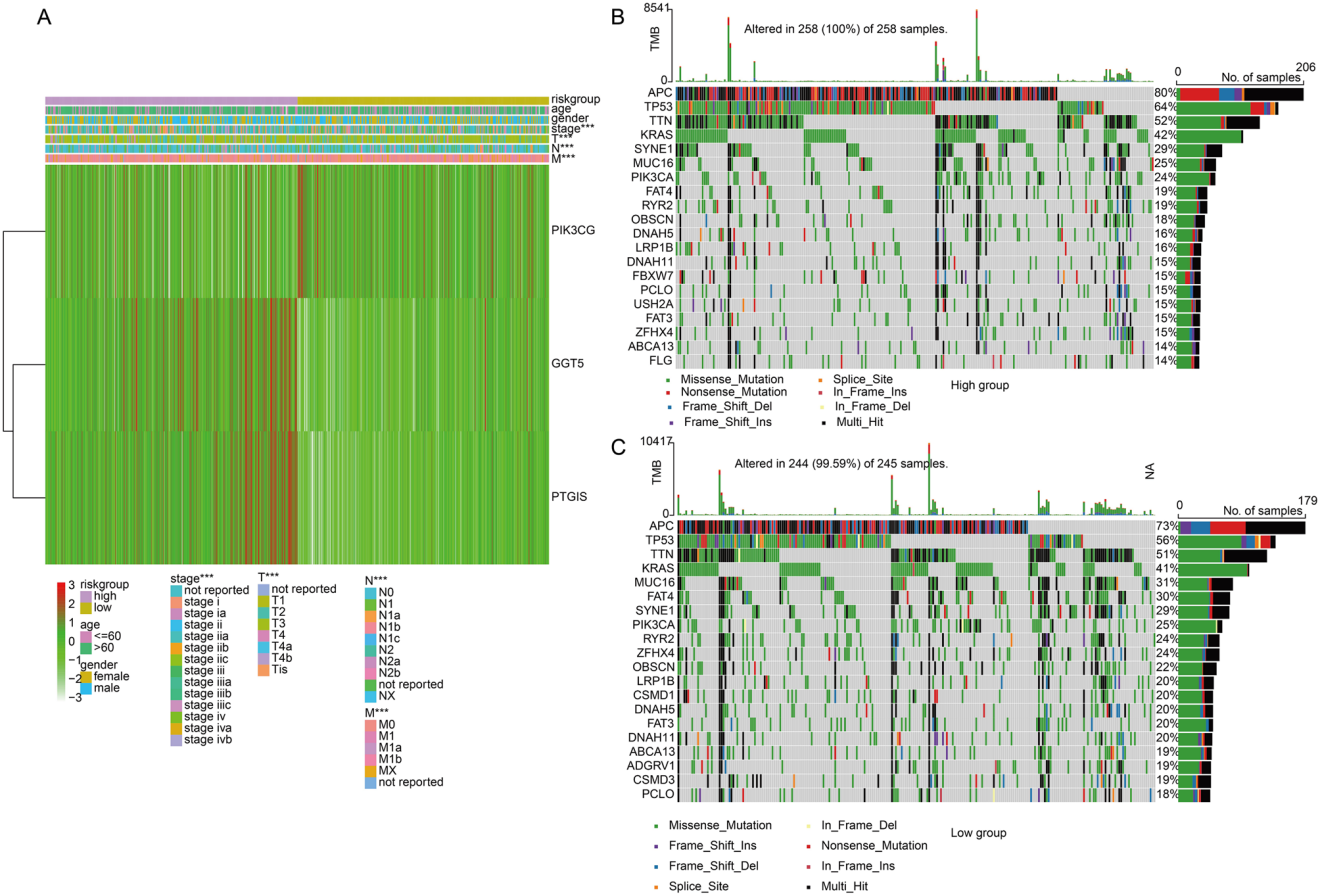
Analysis of mutation characteristics between risk groups in TCGA dataset. (**A**) Distributions of clinicopathologic characteristics between high- and low-risk groups. (**B**,**C**) Mutation characteristics in high- and low-risk groups. ^***^P < 0.001.

Furthermore, the distributions of clinicopathologic characteristics were analyzed between high- and low-risk groups and found that stage, T stage, N stage and M stage were significantly different between the two risk groups (Fig. 4A). Additionally, 100% patients in high-risk group and 99.59% patients in low-risk group exhibited mutation; And compared to low-risk group, patients in high-risk group had a higher genes mutation frequency (APC: 80 vs 73%, TP53: 64 vs 56%, TTN: 52 vs 51%, KRAS: 42 vs 41%) (Fig. 4B,C), indicated the malignancy was higher in the high-risk group.

### Metabolism pathways differences between risk groups

**Figure 5:**
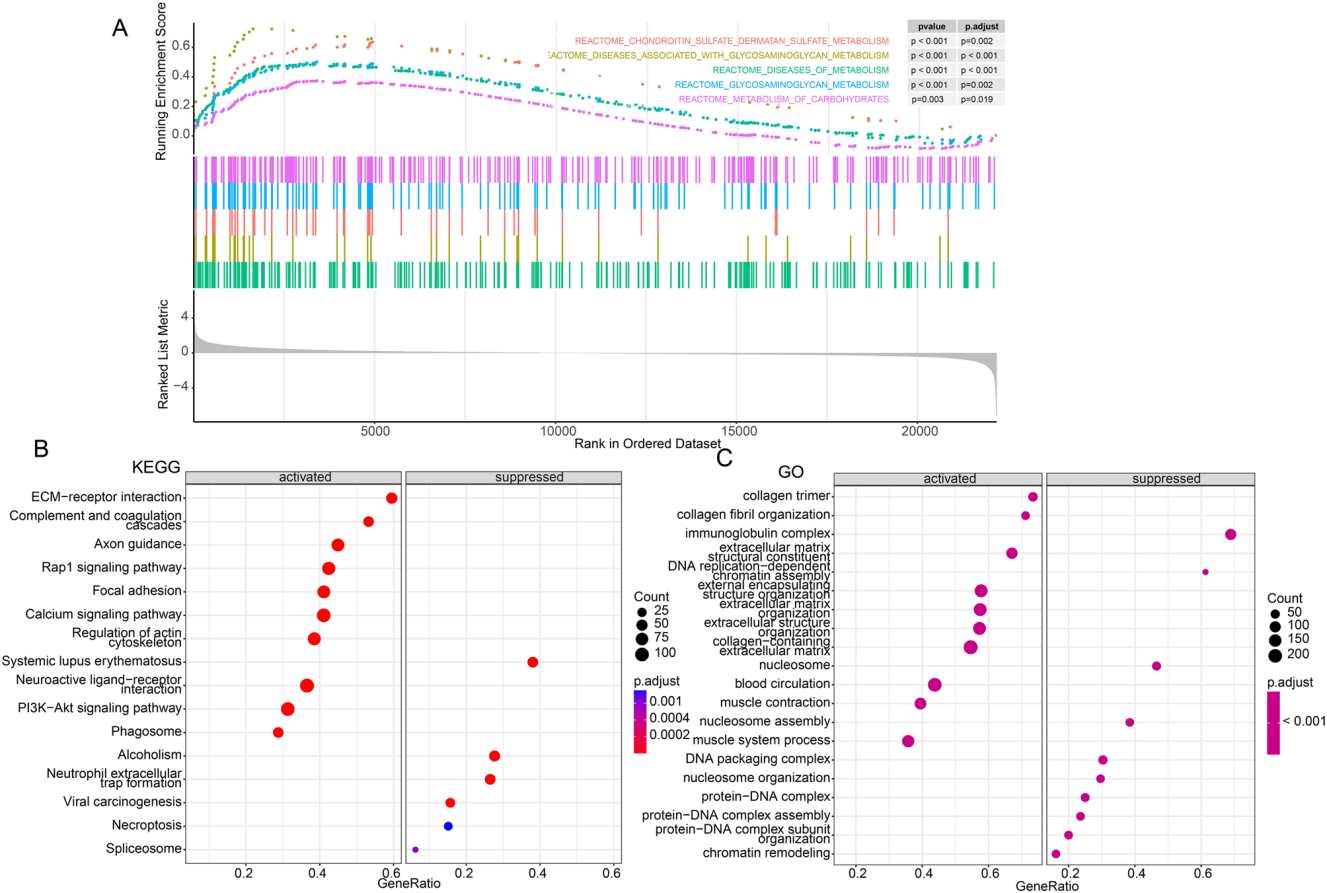
Association of risk score with metabolism pathways in TCGA dataset. (**A**) The results from GSEA showing significant enriched metabolism-related pathways in high risk group. (**B**,**C**) KEGG and GO enrichment analyses using GSEA.

Through GSEA, some metabolism-related pathways such as REACTOME_CHONDROITIN_SULFATE_DERMATAN_SULFATE_METABOLISM (*P* = 9.330e − 05), REACTOME_DISEASES_ASSOCIATED_WITH_GLYCOSAMINOGLYCAN_METABOLISM (*P* = 3.031e − 06), REACTOME_DISEASES_OF_METABOLISM (*P* = 5.284e − 08), REACTOME_GLYCOSAMINOGLYCAN_METABOLISM (*P* = 1.283e − 04) and REACTOME_METABOLISM_OF_CARBOHYDRATES (*P* = 1.558e − 03) were enriched in high-risk group (Fig. 5A). From KEGG analysis, ECM-receptor interaction, focal adhesion, calcium signaling pathway and PI3K-Akt signaling pathway were activated in high-risk group (Fig. 5B); Based on GO analysis, extracellular matrix structural constituent and extracellular matrix organization were activated while DNA replication-dependent chromatin assembly was suppressed in high-risk group (Fig. 5C).

### Analysis of immune characteristics between risk groups

**Figure 6:**
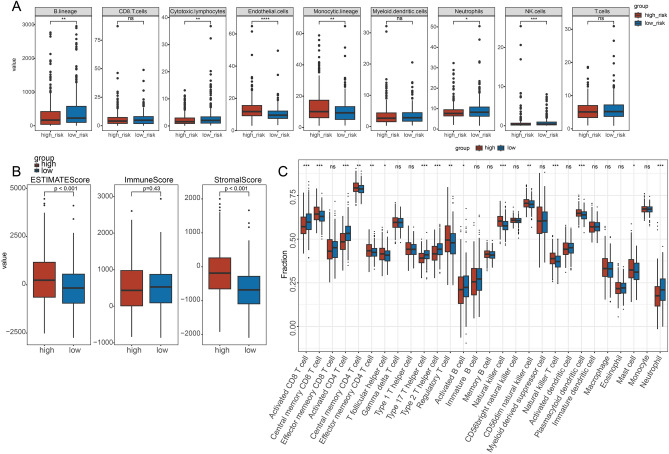
Association of risk score with immune characteristics in TCGA dataset. (**A**) Infiltration of immune cells evaluated by MCPcounter analysis. (**B**) ESTIMATE analysis for evaluating ESTIMATEScore, ImmuneScore and StromalScore. (**C**) SsGSEA scores for 28 immune cells from TISIDB database. Ns represents *P* > 0.05; ^*^P < 0.05, ^**^P < 0.01, and ^***^P < 0.001.

Based on MCPcounter analysis, infiltration of immune cells was significantly varied between the two risk groups. The abundance of endothelial cells and monocytic lineage was increased in patients in high-risk group, while other immune cells including B lineage, CD8 T cells, cytotoxic lymphocytes, neutrophils and NK cells were enriched in patients in low-risk group (Fig. 6A). The results from ESTIMATE analysis showed that patient in high-risk group possessed higher ESTIMATEScore (*P* < 0.001 and StromalScore (*P* < 0.001) than that of patient in low-risk group (Fig. 6B). Meanwhile, the infiltration of central memory CD8 T cell, central memory CD4 T cell, T follicular helper cell, regulatory T cell, natural killer cell, plasmacytoid dendritic cell and mast cell was increased in high-risk patients; activated CD8 T cell, activated CD4 T cell, Type 17 T helper cell, Type 2 T helper cell, activated B cell and neutrophil were abundant in patients in low-risk group (Fig. 6C).

### Prediction of responses to immunotherapy and chemotherapy

**Figure 7:**
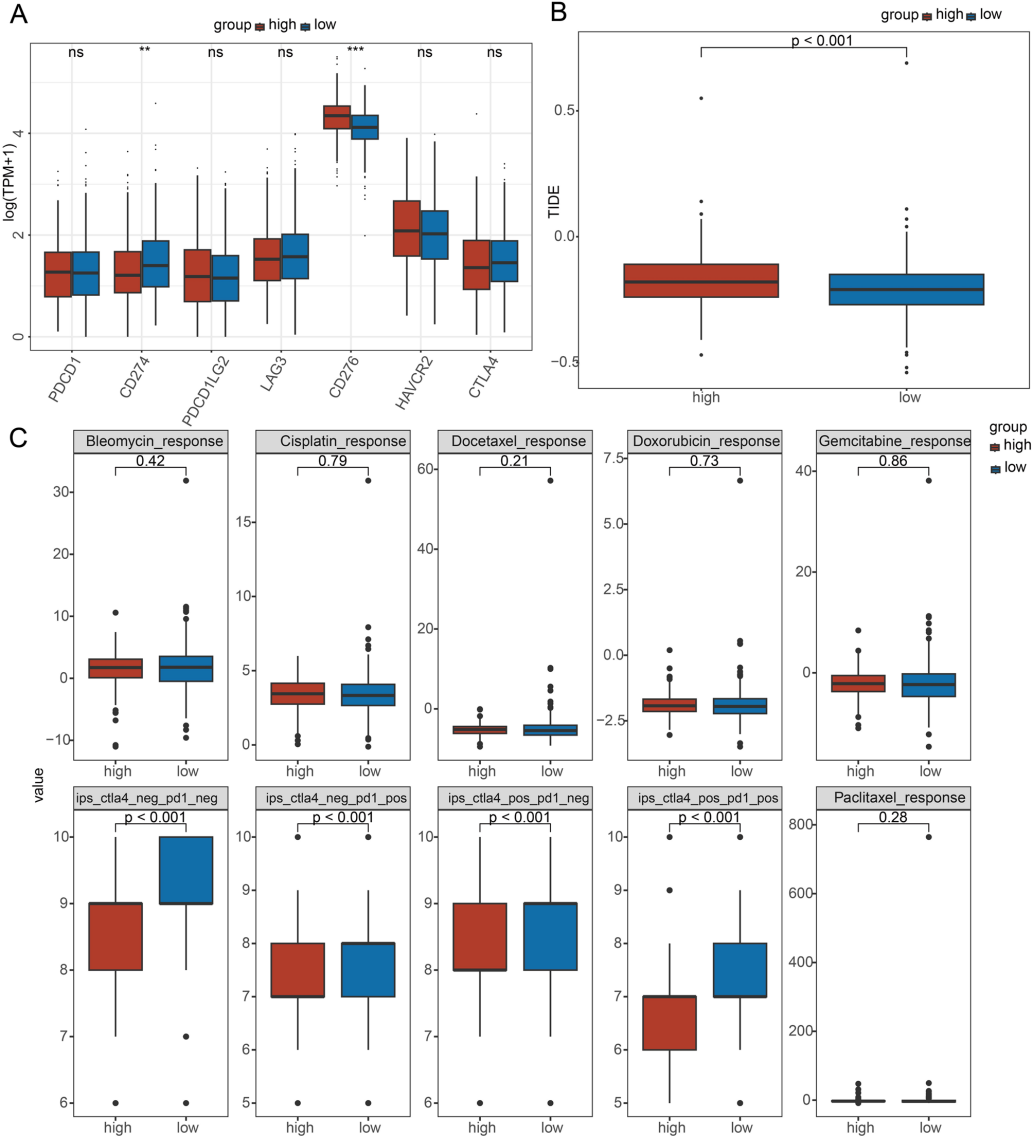
Prediction of responses to immunotherapy and chemotherapy in TCGA dataset. (**A**) Expression of immune checkpoints in high- and low-risk group. (**B**) Changes of TIDE score between high- and low-risk group. (**C**) Estimated IC50 values for traditional chemotherapy drugs. Ns represents *P* > 0.05; ^*^P < 0.05, ^**^P < 0.01, and ^***^P < 0.001.

To predict the response to immunotherapy, the expression of ICs was evaluated in the two risk groups. Patients in low-risk group exhibited higher CD274 level while high-risk group had higher expression of CD276 (Fig. 7A). Figure 7B revealed high-risk group had higher TIDE score than that of low-risk group, indicating a low response rate to ICI therapy of high-risk patients. Additionally, the IPS values for ctla4_neg_pd1_neg (*P* < 0.001), ctla4_neg_pd1_pos (*P* < 0.001), ctla4_pos_pd1_neg (*P* < 0.001) and ctla4_pos_pd1_pos (*P* < 0.001) were higher in low-risk group, which indicated that low-risk group had more immunogenicity on ICIs (Fig. 7C).

### Analysis of risk genes at single cell level

**Figure 8:**
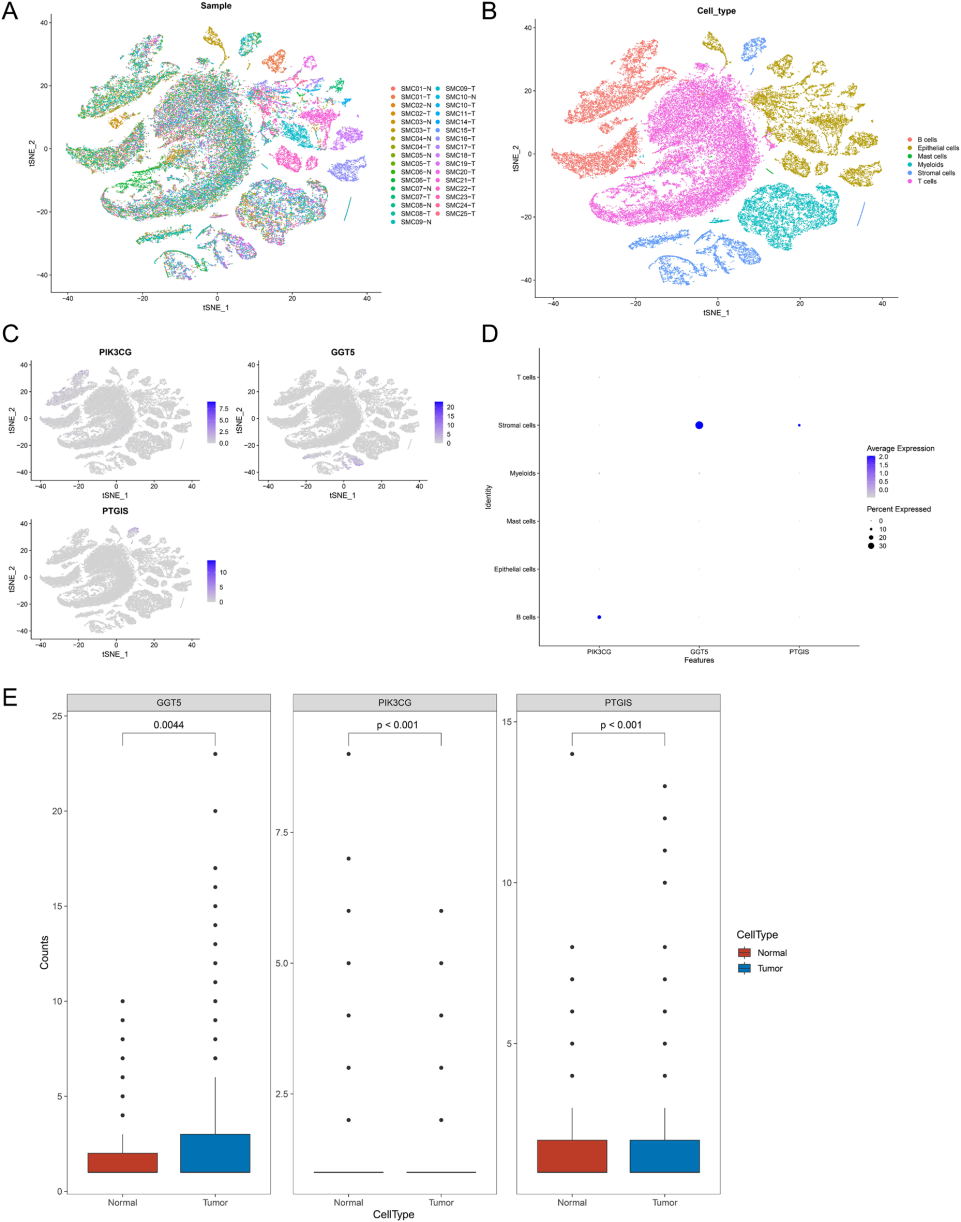
Analysis of risk genes at single cell level. (**A**) TSNE diagrams of 23 CRC samples and 10 healthy samples. (**B**) Screening for 6 cell subpopulations. (**C**,**D**) The distribution of risk genes in 6 cell subpopulations. (**E**) Validation of the expression levels of PIK3CG, GGT5 and PTGIS in single cell data.

Single cell data was acquired for the comprehensively understanding of the profile of risk genes in COAD patients. Figure 8A displayed the overlapped 23 CRC samples and 10 healthy samples in TSNE diagram. 6 cell subpopulations with some classic markers of immune cells such as B cells, ephithelial cells, mast cells, myeloids, stromal cells and T cells were determined (Fig. 8B). Next, the distribution of risk genes in cell subpopulations were evaluated, and PIK3CG was expressed in B cells; GGT5 and PTGIS were both expressed in stromal cells (Fig. 8C,D). Thereafter, the expression levels of PIK3CG, GGT5 and PTGIS were validated in single cell data. The expression of PIK3CG (*P* < 0.001) was downregulated in CRC samples whereas the levels of GGT5 (*P* = 0.0044) and PTGIS (*P* < 0.001) were increased in CRC samples compared with normal samples (Fig. 8E).

## Discussion

Abnormality of lipid metabolism is associated with EMT, cancer stemness and immune checkpoints, which contribute to the metastasis of cancer. Previously, a study used Single sample gene-set enrichment analysis (ssGSEA), univariate, LASSO, and stepwise multiple COX analysis to successfully develop a metabolism-based prognostic risk score for head and neck squamous cell carcinoma^15^. EMT is one of the characteristics of malignant tumors, and many studies have shown that EMT activation is closely related to the production of cancer stem cells in various types of cancer. EMT activation can also enable tumor cells to escape immune killing by increasing the expression of immune checkpoint genes and gain resistance to chemotherapy and immunotherapy, which is also a characteristic of CSCs^16,17^. In this study, we found that primary tumor samples possessed significant increased EMT score and stemness score indicating primary tumor samples maybe presented EMT, acquired characteristics of CSCs and escaped immune killing. Furthermore, 12 lipid metabolism-related genes that were associated with EMT, stemness and checkpoint signatures were identified. Next, a prognostic signature based on PIK3CG, GGT5 and PTGIS was firstly constructed; high-risk group exhibited poor prognosis, higher ESTIMATEScore and StromalScore, 100% mutation rate and higher TIDE score. low-risk group had more immunogenicity on ICIs by evaluating the IPS for CTLA4 and PD-1. Intriguingly, the distribution of risk genes in cell subpopulations were evaluated at single cell level and found that PIK3CG was expressed in B cells; GGT5 and PTGIS were both expressed in stromal cells. This study shed novel insights into the underlying mechanism for COAD in terms of lipid metabolism-related and generated prognostic signatures in predicting prognosis and immunotherapy response.

Lv has recognized that TP53, KRAS and APC are the most frequently earliest somatic mutations, and they are regarded as driver events for CRC; meanwhile other mutations such as CSMD3, TTN and ERBB4 as the latest mutations may contribute to CRC progression^18^. Notably, KRAS are frequently co-mutated with APC (80%) and with TP53 (40%) in CRC^19^. The mutation of KRAS is related to immunosuppression and immune infiltration^20^. TP53 and APC are tumor suppressor genes that guide the use of cetuximab in CRC. Zhang and colleagues have demonstrated that APC mutation was observed in 80% samples in metabolism-associated subtype C1, 67% in subtype C2 and 59% in subtype C3, which may prevent choosing resistant chemotherapeutic drugs for COAD patients with different classification^21^. Additionally, TTN, SYNE1, MUC16, OBSCN and FAT4 are highly mutated in amino acid metabolism-related AA1 subtype exhibiting poor prognosis. In the present study, APC, TP53, TTN, KRAS, SYNE1 and MUC16 were the top 6 mutated genes in high-risk group, APC, TP53, TTN, KRAS, MUC16 and FAT4 were highly mutated in low-risk group, which facilitate prognosis prediction and the decision-making of chemotherapeutic or immunotherapeutic therapies.

The interaction between tumor cells and immune response within the TME affects the evolution of cancer. Moreover, understanding the characteristics of TME is necessary for immunotherapy. Regulatory T cells (Tregs) can serve as antitumorigenic or pro-tumorigenic cells for CRC majorly depending on Treg subsets with diverse immunosuppressive molecules^22^. Syed Khaja has unveiled that CD4 + FoxP3 + Helios + Tregs with highly expressed PD-1/CTLA-4 and PD-1/CD39 suppresses the activation and function of T cells, which provides a promising approach for increasing immune response to dampen CRC^22^. Endothelial cells are one of the main cell types of stromal cells and participate in immunosuppressive TME through release of TGF-β or reducing major histocompatibility class II and leukocyte adhesion molecules in CRC^23^. The aggression of CRC is closely related to mast cells around the tumor periphery along with the inflammatory cells. Besides, infiltration of mast cells enhances the resistance to anti-PD-1/PD-L1 immunotherapy^24^. Li has revealed that PD-1 antibody induces the activation of PD-1 + mast cells through via releasing histamine and cytokines from mast cell and application of cromolyn sodium inhibits mast cell infiltration. Therefore, inhibition of mast cell infiltration may overcome the unfavorable response to anti-PD-1 blockade^25^. In this study, endothelial cells, Tregs and mast cells were abundant in COAD patients with high risk, indicating tumor-infiltration of these immune cells contributed to COAD development, whereas B cells, CD8 T cells, cytotoxic lymphocytes and NK cells eliminated tumor cells. Meanwhile, immunotherapies seeking to inhibit infiltration of endothelial cells, Tregs and mast cells but activate cytotoxic immune cells are benefit to COAD.

Furthermore, CD276 gene encoding B7-H3 was highly expressed in high-risk group, while CD274 encoding PD-L1 was increased in low-risk group. B7-H3 has been regarded as a promising pan-cancer target. However, Zhang has deciphered that the heterogeneity and variation are frequently occurred for B7-H3 expression, which may lead to the difficult for clinical practice^26^. Moreover, the variant expression of CD276 and CD274 between high- and low-risk group might contribute to different TME and therapy response, which should be carefully considered in immunotherapies for COAD. Furthermore, the response of COAD patients to immunotherapy was evaluated and found that low-risk group exhibited higher IPS for ctla4_neg_pd1_neg, ctla4_neg_pd1_pos, ctla4_pos_pd1_neg and ctla4_pos_pd1_pos, implying that lipid metabolism-based risk score possessed potentials to determine the response to ICI therapies for the specific COAD patients.

Firstly, based on 742 lipid metabolism-related genes, WGCNA identified green module and yellow module were closely correlation between and EMT, ICs, stemness. Under |kME|> 0.8 condition, 12 key genes were obtained from green module and yellow module. LASSO was further used to compress key genes, and when optimal lambda = 0.01463613, Three genes (PIK3CG, GGT5 and PTGIS) were used to construct the prognostic model. Additionally, scRNA-seq analysis showed that PIK3CG was expressed in B cells; GGT5 and PTGIS were both expressed in stromal cells. PIK3CG encodes phosphatidylinositol 3-kinase γ and it plays crucial roles in COAD. A findings suggest that the silencing of the PIK3CG gene inhibited the PI3K-Akt/PKB signaling system responsible for tumorigenesis and the progression of colorectal cancers^27^. A recent spatial transcriptomic analysis have deciphered that monocytoid cells are recognized as atypical memory B cells exhibiting downregulated PIK3CG associated with the activation of B cells^28^. It has been demonstrated that PIK3CG is expressed in B and T cells while it is less expressed than HCK in myeloid cells based on scRNA-seq analysis of MC38 tumors^29^. These findings imply that PIK3CG may be as B cell-specific drug target for COAD. PTGIS is a prostacyclin synthase and is highly expressed in colon cancer liver metastasis^30^. One study found that the expression of PTGIS gene in colorectal cancer tissues was significantly lower than that in normal colorectal cancer tissues, and the high expression of PTGIS gene was associated with poor prognosis of patients^31^. Previous study has confirmed stromal cells can induce the increased expression of PTGIS and PGI2, which may lead to tumor cell growth and unfavorable outcomes^32,33^. Gamma-glutamyltransferase 5 (GGT5) belongs to GGT family possessing catalytic activity. Recently, Jun Ren and colleagues have identified that GGT5 as one of lipid metabolism-related genes for the prediction of COAD prognosis^34^, whereas they did not elucidate the response to immunotherapy/chemotherapy and the distribution of GGT5 in stromal cells at single cell level. A study reported that GGT5 is an independent prognostic biomarker in stomach adenocarcinoma^35^. In this study, lipid metabolism-related genes correlated with EMT, stemness and immune checkpoint signatures was firstly identified; also, the cell subpopulations that lipid metabolism-related genes expressed in, which might offer the potential cell subpopulations-specific drug targets for COAD management.

Limitations should be noted in this study. Although three risk genes were determined, retrospective data used in this study are collected from TCGA database; further prospective studies with large samples are necessary to validate our results. The reliability of this prognostic signature should be iteratively improved with long-term clinical application. Besides, PIK3CG was expressed in B cells, while GGT5 and PTGIS were both expressed in stromal cells, and the expression levels of PIK3CG, GGT5 and PTGIS were validated in single cell data; however, the underlying mechanism of PIK3CG, GGT5 and PTGIS contributing to heterogeneity of COAD remains unclarified. Therefore, experimental studies should be performed to reveal their regulatory effects.

## Conclusion

To sum up, a metabolic gene co-expression network for COAD and identified metabolic genes associated with EMT, stemness and immune checkpoint signatures was generated. Next, lipid metabolism-based prognostic signature was constructed to forecast the prognosis, immunotherapy and chemotherapy response in COAD patients. PIK3CG was expressed in B cells, while GGT5 and PTGIS were expressed in stromal cells, which might reveal the potential mechanism for heterogeneity of COAD and provide a meaningful guidance for therapeutics development.

## Material and methods

### Data collection

The transcriptome data and clinical information of colon and rectal adenocarcinoma (COAD and READ) were collected from The Cancer Genome Atlas (TCGA) database (https://portal.gdc.cancer.gov/). The TCGA-COAD and TCGA-READ were combined and a total of 675 COAD samples were obtained in this study. Samples lacking clinical follow-up information, survival time and survival states were excluded, and then Ensembl gene IDs were converted to Gene symbol IDs. The single-cell RNA sequencing (scRNA-seq) expression profiling in GSE132465 dataset^23^ was downloaded from Gene-Expression Omnibus (GEO; https://www.ncbi.nlm.nih.gov/geo/) database, including 23 colorectal cancer (CRC) samples and 10 healthy samples. Seurat R package^36^ was applied to analyze the data of 65,362 cells in GSE132465 dataset and annotated 6 cell subpopulation.

### Single sample gene set enrichment analysis (ssGSEA)

A set of epithelial-mesenchymal transition (EMT) genes including 1184 genes were collected from the DbEMT database that is used to explore EMT-related human genes^37^. Stem cell-related gene set containing 4419 genes were obtained from the StemChecker database^38^. Besides, a total of 79 immune checkpoint (IC) genes were downloaded as previously reported^39^. In order to investigate the differences of EMT-, stem cell-, and IC-related genes between COAD and control samples, ssGSEA was performed in TCGA dataset, and box plots were used for visualization.

### Weighted gene co-expression network analysis (WGCNA)

Next, lipid metabolism-related gene set comprising 742 genes (REACTOME_METABOLISM_OF_LIPIDS_genes) were downloaded from the Molecular Signatures Database (MSigDB, https://www.gsea-msigdb.org/gsea/msigdb). Based on the aforementioned lipid metabolism-related pathway genes, the co-expressed genes and co-expression modules were determined using “WGCNA” R package (version1.72-5, http://horvath.genetics.ucla.edu/html/CoexpressionNetwork/Rpackages/WGCNA/)^40^. It is worth mentioning that there is no clear threshold for removing outliers, but in principle, the branches after sample clustering should be as concise as possible while reflecting the characteristics of the sample. The Pearson correlation coefficient was calculated to assess the similarity of the gene expression profiling. The correlation matrix was converted into adjacency matrix. To ensure the co-expression network conforms to the principle of scale-free distribution, R^2 > 0.8 was considered as a threshold to determine the soft threshold value (power = 4). Subsequently, the relationships between modules and signatures (EMT, ICs and stemness) were calculated in order to identify the gene set modules highly related to signatures. To identify hub genes within modules highly related to signatures, the intramodular connectivity |kME|> 0.8 was regarded as the threshold.

### Generation of prognostic model and survival analysis

Based on hub genes identified through WGCNA, the least absolute shrinkage and selection operator (LASSO) Cox regression was performed using “glmnet” package^41^ in R. Five-fold cross-validation was conducted to minimize the partial likelihood deviance error. The risk score formula related to the prognostic signatures was as follows:

$$ {\text{Risk score}} = \sum {\beta i} \times {\text{ExPi,}} $$

where the “β” represents the Cox regression coefficient value and "ExPi" indicates the i gene expression. The risk score was calculated for each sample in TCGA dataset, and samples in TCGA dataset were divided into high-risk group and low-risk group based on the median value. Samples with risk score > media value were put into high-risk group, while samples with risk score < media value were put into low-risk group. Kaplan–Meier curves were generated between high- and low-risk groups. Then, the receiver operating characteristic (ROC) analysis was conducted using “timeROC” package^42^, along with areas under the ROC curve (AUCs) for 1, 3 and 5 years.

### Clinicopathologic features and mutation characteristic analysis between risk groups

The distributions of clinicopathologic characteristics (age, gender, stage, T stage, N stage and M stage) as well as risk genes were compared between high- and low-risk groups. Furthermore, the single nucleotide variations (SNVs) data were downloaded from TCGA through mutect2 software and genes with mutation frequency > 3 were selected for further analysis. “maftools” package^43^ was utilized to analyze SNVs data in high- and low-risk groups.

### Metabolism pathways differences between risk groups

Metabolism-related pathways were downloaded from Reactome Pathway Database (https://reactome.org/) using “msigdbr” package^44^ and GSEA was used to evaluate the differences of metabolism pathway characteristics between the two risk groups. Also, the Kyoto Encyclopedia of Genes and Genomes (KEGG) and gene ontology (GO) enrichment analyses were performed in this study.

### Analysis of immune characteristics between low- and high-risk groups

ESTIMATEScore, ImmuneScore and StromalScore were evaluated in TCGA dataset using ESTIMATE algorithm. Besides, the microenvironment cell populations-counter (MCP-counter) method^45^ was employed to assess the infiltration of immune cells in risk groups. Totally, 28 immune cells were also obtained from TISIDB database^46^ and ssGSEA was applied to score the immune cells.

### Immunotherapy and chemotherapy response

The tumor immune dysfunction exclusion (TIDE) algorithm (http://tide.dfci.harvard.edu/) was used to evaluate the response to immune checkpoint inhibition (ICI) therapy. A high TIDE score indicates a low response rate to ICI therapy. Meanwhile, immune checkpoints (ICs) were obtained from a previous study^47^. The expression of ICs was analyzed between high- and low-risk groups. The clinical response to ICI therapy (PD-1 and CTAL4) was evaluated by IPS.

### Single cell analysis

In order to evaluate the expression of risk genes at single cell level, scRNA-seq data was acquired and analyzed the expression patterns of risk genes (PIK3CG, GGT5 and PTGIS) in normal and tumor samples. The t-distributed stochastic neighbor embedding (TSNE) was generated using RunTSNE founction and cell subpopulations with some classic markers of immune cells were then annotated.

### Statistical analyses

Data analyses were conducted using R package (version 3.6.3, https://www.r-project.org/ ver. 3.6.3). Wilcox.tests was applied to analyze differences between high- and low-risk groups. Chi square test was use to compare the distribution of clinicopathologic characteristics between risk groups. Log-rank test was used to determine the significance of differences for Kaplan–Meier curves. Fisher tests were used to select highly mutated genes in high- and low-risk groups. *P* < 0.05 was considered statistically significant.

## Supplementary Information

Supplementary Figure 1.

Supplementary Figure 2.

Supplementary Legends.

## Data Availability

The datasets generated during and/or analysed during the current study are available from the corresponding author on reasonable request.

## References

[1] Sung, H. *et al.* Global cancer statistics 2020: GLOBOCAN estimates of incidence and mortality worldwide for 36 cancers in 185 countries. *CA Cancer J. Clin.* **71**(3), 209–249 (2021). 33538338 10.3322/caac.21660

[2] Xi, Y. & Xu, P. Global colorectal cancer burden in 2020 and projections to 2040. *Transl. Oncol.* **14**(10), 101174 (2021). 34243011 10.1016/j.tranon.2021.101174 PMC8273208

[3] Fleming, M. *et al.* Colorectal carcinoma: Pathologic aspects. *J. Gastrointest. Oncol.* **3**(3), 153 (2012). 22943008 10.3978/j.issn.2078-6891.2012.030 PMC3418538

[4] Sagaert, X., Vanstapel, A. & Verbeek, S. Tumor heterogeneity in colorectal cancer: What do we know so far?. *Pathobiology* **85**(1–2), 72–84 (2018). 29414818 10.1159/000486721

[5] Hu, L.-F. *et al.* Personalized immunotherapy in colorectal cancers: Where do we stand?. *Front. Oncol.* **11**, 769305 (2021). 34888246 10.3389/fonc.2021.769305 PMC8649954

[6] Yeung, K. T. & Yang, J. Epithelial–mesenchymal transition in tumor metastasis. *Mol. Oncol.* **11**(1), 28–39 (2017). 28085222 10.1002/1878-0261.12017 PMC5242415

[7] Mortezaee, K., Majidpoor, J. & Kharazinejad, E. Epithelial-mesenchymal transition in cancer stemness and heterogeneity: Updated. *Med. Oncol.* **39**(12), 193 (2022). 36071302 10.1007/s12032-022-01801-0

[8] Humphries, H. N. *et al.* Characterization of cancer stem cells in colon adenocarcinoma metastasis to the liver. *Front. Surg.* **4**, 76 (2018). 29404335 10.3389/fsurg.2017.00076 PMC5786574

[9] Xu, F. *et al.* Cancer stemness, immune cells, and epithelial–mesenchymal transition cooperatively predict prognosis in colorectal carcinoma. *Clin. Colorectal Cancer* **17**(3), e579–e592 (2018). 29921496 10.1016/j.clcc.2018.05.007

[10] Kang, H. *et al.* Role of metabolic reprogramming in epithelial–mesenchymal transition (EMT). *Int. J. Mol. Sci.* **20**(8), 2042 (2019). 31027222 10.3390/ijms20082042 PMC6514888

[11] Tian, W. *et al.* FABP4 promotes invasion and metastasis of colon cancer by regulating fatty acid transport. *Cancer Cell Int.* **20**, 1–13 (2020). 33088219 10.1186/s12935-020-01582-4 PMC7574203

[12] Zhang, C. *et al.* Cancer-derived exosomal HSPC111 promotes colorectal cancer liver metastasis by reprogramming lipid metabolism in cancer-associated fibroblasts. *Cell Death Dis.* **13**(1), 57 (2022). 35027547 10.1038/s41419-022-04506-4 PMC8758774

[13] Li, H., Feng, Z. & He, M. L. Lipid metabolism alteration contributes to and maintains the properties of cancer stem cells. *Theranostics* **10**(16), 7053 (2020). 32641978 10.7150/thno.41388 PMC7330842

[14] Yi, M. *et al.* Emerging role of lipid metabolism alterations in cancer stem cells. *J. Exp. Clin. Cancer Res.* **37**(1), 1–18 (2018). 29907133 10.1186/s13046-018-0784-5 PMC6003041

[15] Xing, L. *et al.* A transcriptional metabolic gene-set based prognostic signature is associated with clinical and mutational features in head and neck squamous cell carcinoma. *J. Cancer Res. Clin. Oncol.* **146**(3), 621–630 (2020). 32067104 10.1007/s00432-020-03155-4 PMC11804320

[16] Tsuchiya, H. & Shiota, G. Immune evasion by cancer stem cells. *Regen. Ther.* **17**, 20–33 (2021). 33778133 10.1016/j.reth.2021.02.006 PMC7966825

[17] Najafi, M., Farhood, B. & Mortezaee, K. Cancer stem cells (CSCs) in cancer progression and therapy. *J. Cell Physiol.* **234**(6), 8381–8395 (2019). 30417375 10.1002/jcp.27740

[18] Lv, J. *et al.* Sclonal architectures predict clinical outcome in colon adenocarcinoma. *J. Cell. Mol. Med.* **25**(3), 1796 (2021). 33369051 10.1111/jcmm.16208 PMC7875899

[19] Timar, J. & Kashofer, K. Molecular epidemiology and diagnostics of KRAS mutations in human cancer. *Cancer and Metastas. Rev.* **39**, 1029–1038 (2020). 10.1007/s10555-020-09915-5 PMC7680318 32725342

[20] Liu, J. *et al.* Immune landscape and prognostic immune-related genes in KRAS-mutant colorectal cancer patients. *J. Transl. Med.* **19**, 1–17 (2021). 33413474 10.1186/s12967-020-02638-9 PMC7789428

[21] Zhang, M. *et al.* Metabolism-associated molecular classification of colorectal cancer. *Front. Oncol.* **10**, 602498 (2020). 33344254 10.3389/fonc.2020.602498 PMC7746835

[22] Syed Khaja, A. S. *et al.* Intratumoral FoxP3+ Helios+ regulatory T cells upregulating immunosuppressive molecules are expanded in human colorectal cancer. *Front. Immunol.* **8**, 619 (2017). 28603527 10.3389/fimmu.2017.00619 PMC5445103

[23] Lee, H.-O. *et al.* Lineage-dependent gene expression programs influence the immune landscape of colorectal cancer. *Nat. Genet.* **52**(6), 594–603 (2020). 32451460 10.1038/s41588-020-0636-z

[24] Kwantwi, L. B. Overcoming anti-PD-1/PD-L1 immune checkpoint blockade resistance: The role of macrophage, neutrophils and mast cells in the tumor microenvironment. *Clin. Exp. Med.* **23**, 1–15 (2023). 37022584 10.1007/s10238-023-01059-4

[25] Li, J. *et al.* PD-1+ mast cell enhanced by PD-1 blocking therapy associated with resistance to immunotherapy. *Cancer Immunol. Immunother.* **72**(3), 633–645 (2023). 36018370 10.1007/s00262-022-03282-6 PMC9947072

[26] Zhang, Z. *et al.* B7-H3-targeted CAR-T cells exhibit potent antitumor effects on hematologic and solid tumors. *Mol. Ther. Oncolytics* **17**, 180–189 (2020). 32346608 10.1016/j.omto.2020.03.019 PMC7178328

[27] Semba, S. *et al.* Down-regulation of PIK3CG, a catalytic subunit of phosphatidylinositol 3-OH kinase, by CpG hypermethylation in human colorectal carcinoma. *Clin. Cancer Res.* **8**(12), 3824–3831 (2002). 12473596

[28] Yu, S. C., Chen, K. C. & Huang, R. Y. J. Nodal reactive proliferation of monocytoid B-cells may represent atypical memory B-cells. *J. Microbiol. Immunol. Infect.* **56**(4), 729–738 (2023). 37080839 10.1016/j.jmii.2023.03.010

[29] Poh, A. R. *et al.* Therapeutic inhibition of the SRC-kinase HCK facilitates T cell tumor infiltration and improves response to immunotherapy. *Sci. Adv.* **8**(25), eab17882 (2022). 10.1126/sciadv.abl7882 PMC9216510 35731867

[30] Lichao, S. *et al.* Overexpression of PTGIS could predict liver metastasis and is correlated with poor prognosis in colon cancer patients. *Pathol. Oncol. Res.* **18**(3), 563–569 (2012). 22109564 10.1007/s12253-011-9478-4

[31] Ding, H. *et al.* Validating the role of PTGIS gene in colorectal cancer by bioinformatics analysis and in vitro experiments. *Sci. Rep.* **13**(1), 16496 (2023). 37779109 10.1038/s41598-023-43289-2 PMC10543560

[32] Lichao, S. *et al.* Overexpression of PTGIS could predict liver metastasis and is correlated with poor prognosis in colon cancer patients. *Pathol. Oncol. Res.* **18**, 563–569 (2012). 22109564 10.1007/s12253-011-9478-4

[33] Sommerfeld, L. *et al.* Prostacyclin released by cancer-associated fibroblasts promotes immunosuppressive and pro-metastatic macrophage polarization in the ovarian cancer microenvironment. *Cancers* **14**(24), 6154 (2022). 36551640 10.3390/cancers14246154 PMC9776493

[34] Ren, J. *et al.* Development and validation of a metabolic gene signature for predicting overall survival in patients with colon cancer. *Clin. Exp. Med.* **20**(4), 535–544 (2020). 32772211 10.1007/s10238-020-00652-1

[35] Huang, Y. *et al.* GGT5 Is an independent prognostic biomarker in stomach adenocarcinoma. *Can. J. Gastroenterol. Hepatol.* **2022**, 9983351 (2022). 35257007 10.1155/2022/9983351 PMC8898138

[36] Gribov, A. *et al.* SEURAT: Visual analytics for the integrated analysis of microarray data. *BMC Med. Genom.* **3**, 1–6 (2010). 10.1186/1755-8794-3-21 PMC2893446 20525257

[37] Zhao, M. *et al.* dbEMT: An epithelial-mesenchymal transition associated gene resource. *Sci. Rep.* **5**(1), 11459 (2015). 26099468 10.1038/srep11459 PMC4477208

[38] Pinto, J. P. *et al.* StemChecker: A web-based tool to discover and explore stemness signatures in gene sets. *Nucleic Acids Res.* **43**(W1), W72–W77 (2015). 26007653 10.1093/nar/gkv529 PMC4489266

[39] Hu, F. F. *et al.* Expression profile of immune checkpoint genes and their roles in predicting immunotherapy response. *Brief. Bioinform.* **22**(3), 176 (2021). 10.1093/bib/bbaa176 32814346

[40] Langfelder, P. & Horvath, S. WGCNA: An R package for weighted correlation network analysis. *BMC Bioinform.* **9**(1), 1–13 (2008). 10.1186/1471-2105-9-559 PMC2631488 19114008

[41] Hastie, T., Qian, J. & Tay, K. An introduction to glmnet. *CRAN R Repos.* **5**, 1–35 (2021).

[42] Blanche, P., Dartigues, J. F. & Jacqmin-Gadda, H. Estimating and comparing time-dependent areas under receiver operating characteristic curves for censored event times with competing risks. *Stat. Med.* **32**(30), 5381–5397 (2013). 10.1002/sim.5958 24027076

[43] Mayakonda, A. *et al.* Maftools: Efficient and comprehensive analysis of somatic variants in cancer. *Genome Res.* **28**(11), 1747–1756 (2018). 30341162 10.1101/gr.239244.118 PMC6211645

[44] Liberzon, A. *et al.* The molecular signatures database hallmark gene set collection. *Cell Syst.* **1**(6), 417–425 (2015). 26771021 10.1016/j.cels.2015.12.004 PMC4707969

[45] Becht, E. *et al.* Estimating the population abundance of tissue-infiltrating immune and stromal cell populations using gene expression. *Genome Biol.* **17**(1), 1–20 (2016). 27908289 10.1186/s13059-016-1113-y PMC5134277

[46] Ru, B. *et al.* TISIDB: An integrated repository portal for tumor–immune system interactions. *Bioinformatics* **35**(20), 4200–4202 (2019). 30903160 10.1093/bioinformatics/btz210

[47] Auslander, N. *et al.* Robust prediction of response to immune checkpoint blockade therapy in metastatic melanoma. *Nat. Med.* **24**(10), 1545–1549 (2018). 30127394 10.1038/s41591-018-0157-9 PMC6693632

