# Author Correction: Generation of novel lipid metabolism-based signatures to predict prognosis and immunotherapy response for colorectal adenocarcinoma

**DOI:** 10.1038/s41598-024-72216-2

**Published:** 2024-09-11

**Authors:** Yi Wang, Jun Yao, Zhe Zhang, Luxin Wei, Sheng Wang

**Affiliations:** 1 Department of Oncology and Hematology, Suzhou Kowloon Hospital, Shanghai Jiao Tong University School of Medicine, Suzhou, 215127 China; 2 Department of General Surgery, The Fourth Affiliated Hospital of Soochow University, Suzhou, 215127 China

Correction to: *Scientific Reports* 10.1038/s41598-024-67549-x, published online 26 July 2024

In the original version of this Article, a Funding information section has been removed.

The original Article has been corrected.

