# Supplementary figures and images for: Generation of novel lipid metabolism-based signatures to predict prognosis and immunotherapy response for colorectal adenocarcinoma
Source: Sci Rep. 2024 Jul 26;14:17158. doi: 10.1038/s41598-024-67549-x (PMC11282063; doi:10.1038/s41598-024-67549-x)

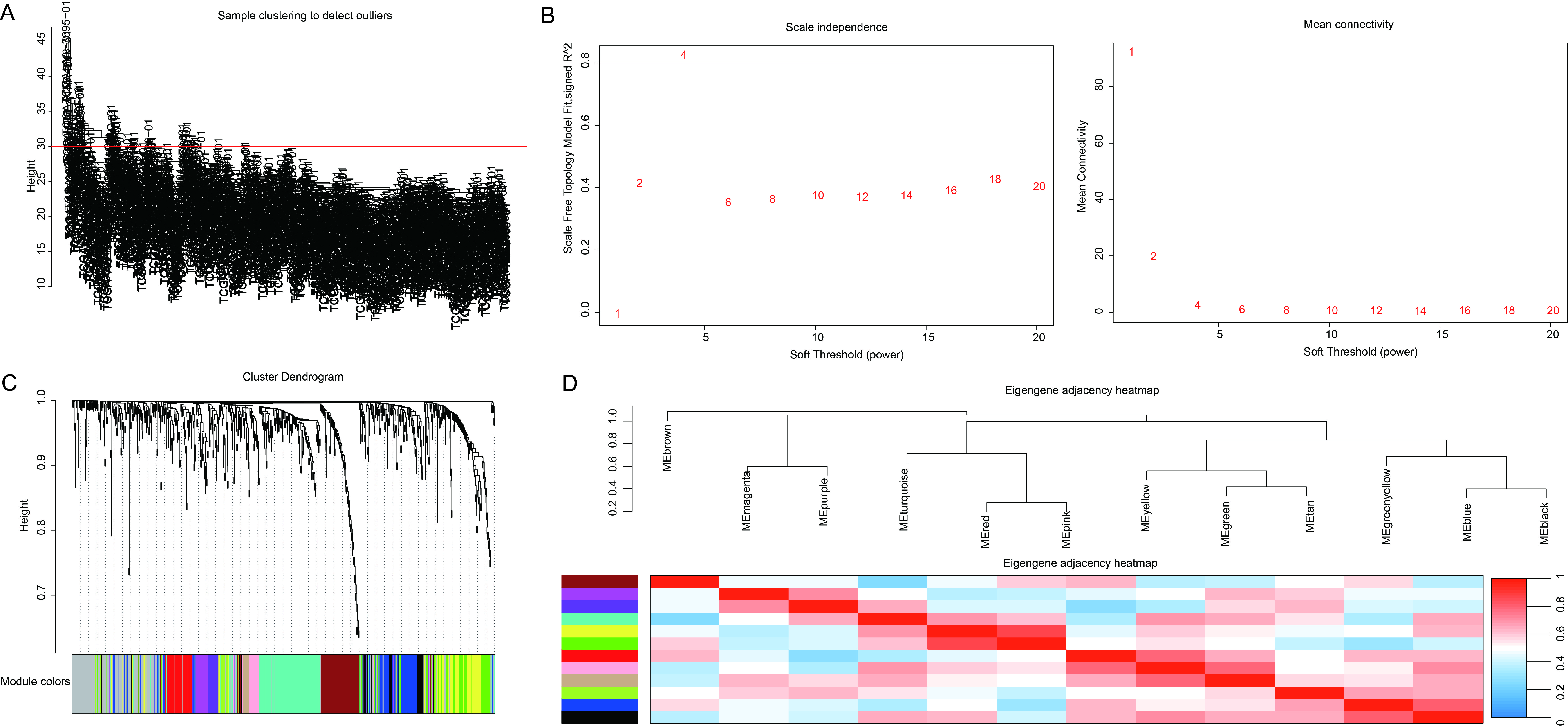

Supplement: Supplementary file 1 — Supplementary Figure 1. [file 41598_2024_67549_MOESM1_ESM.jpg]

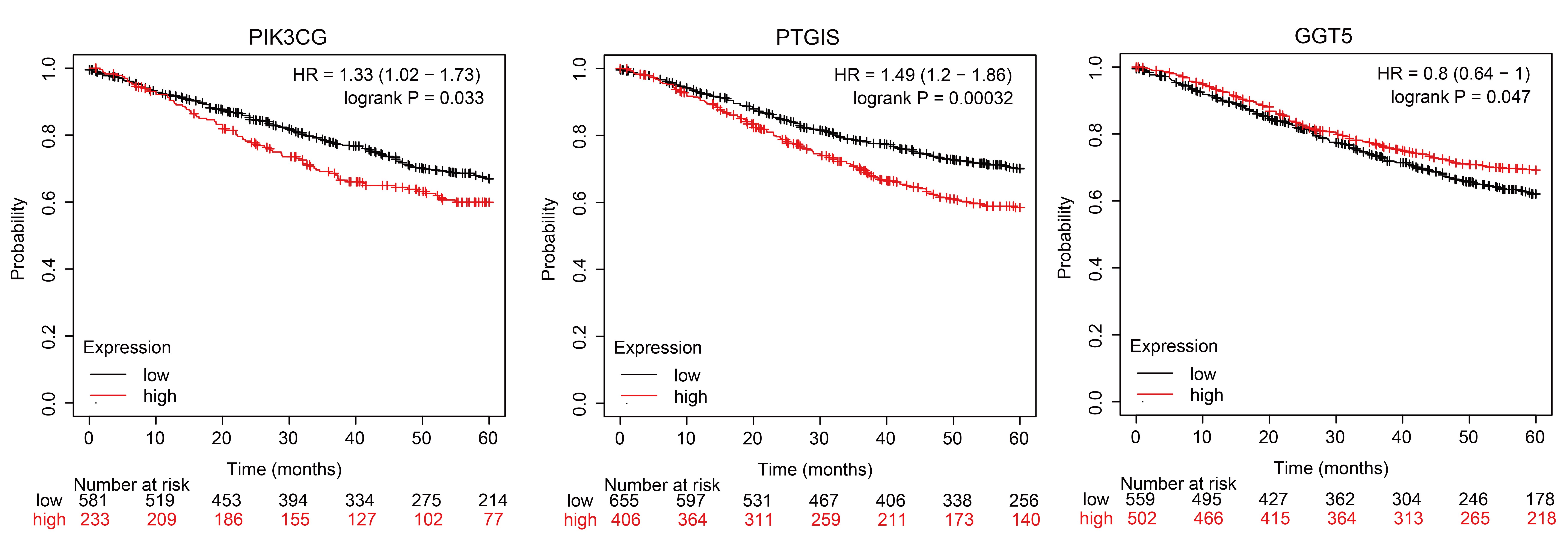

Supplement: Supplementary file 2 — Supplementary Figure 2. [file 41598_2024_67549_MOESM2_ESM.jpg]
