# Supplementary material for: Generation of novel lipid metabolism-based signatures to predict prognosis and immunotherapy response for colorectal adenocarcinoma
Source: Sci Rep. 2024 Jul 26;14:17158. doi: 10.1038/s41598-024-67549-x (PMC11282063; doi:10.1038/s41598-024-67549-x)
Supplement: Supplementary file 3 — Supplementary Legends. [file 41598_2024_67549_MOESM3_ESM.docx]

**Supplementary Figure 1** Identification of co-expression modules and hub genes using WGCNA. A, Sample clustering to detect outliers based on the height cutoff value of 30. B, The soft threshold power is set at 4 with an R^2 > 0.8. C, Cluster dendrogram for co-expressed modules. D, Eigengene adjacency heatmap for 12 modules. WGCNA package (version 1.72-5, <http://horvath.genetics.ucla.edu/html/CoexpressionNetwork/Rpackages/WGCNA/>) was used to created co-expression modules.

**Supplementary Figure 2** KM survival curve of PIK3CG, PTGIS and GGT5 for COAD in KMPLOT database.
